# Lessons From the First Wave of COVID-19: Work-Related Consequences, Clinical Knowledge, Emotional Distress, and Safety-Conscious Behavior in Healthcare Workers in Switzerland

**DOI:** 10.3389/fpsyg.2021.628033

**Published:** 2021-02-09

**Authors:** Marco Riguzzi, Shkumbin Gashi

**Affiliations:** Careum School of Health, Zürich, Switzerland

**Keywords:** COVID-19, healthcare workers, clinical knowledge, risk perception, mental health, stress, work situation, prevention

## Abstract

The coronavirus disease (COVID-19) imposes an unusual risk to the physical and mental health of healthcare workers and thereby to the functioning of healthcare systems during the crisis. This study investigates the clinical knowledge of healthcare workers about COVID-19, their ways of acquiring information, their emotional distress and risk perception, their adherence to preventive guidelines, their changed work situation due to the pandemic, and their perception of how the healthcare system has coped with the pandemic. It is based on a quantitative cross-sectional survey of 185 Swiss healthcare workers directly attending to patients during the pandemic, with 22% (*n* = 40) of them being assigned to COVID-19-infected patients. The participants answered between 16th June and 15th July 2020, shortly after the first wave of COVID-19 had been overcome and the national government had relaxed its preventive regulations to a great extent. The questionnaire incorporated parts of the “Standard questionnaire on risk perception of an infectious disease outbreak” (version 2015), which were adapted to the case of COVID-19. Clinical knowledge was lowest regarding the effectiveness of standard hygiene (*p* < 0.05). Knowledge of infectiousness, incubation time, and life-threatening disease progression was higher, however still significantly lower than regarding asymptomatic cases and transmission without physical contact (*p* < 0.001). 70% (95%-confidence interval: 64-77%) of the healthcare workers reported considerable emotional distress on at least one of the measured dimensions. They worried significantly more strongly about patients, elderly people, and family members, than about their own health (*p* < 0.001). Adherence to (not legally binding) preventive guidelines by the government displayed patterns such that not all guidelines were followed equally. Most of the participants were faced with a lack of protective materials, personnel, structures, processes, and contingency plans. An increase in stress level was the most prevalent among the diverse effects the pandemic had on their work situation. Better medical equipment (including drugs), better protection for their own mental and physical health, more (assigned) personnel, more comprehensive information about the symptoms of the disease, and a system of earlier warning were the primary lessons to be learned in view of upcoming waves of the pandemic.

## Introduction

**FIGURE 1:**
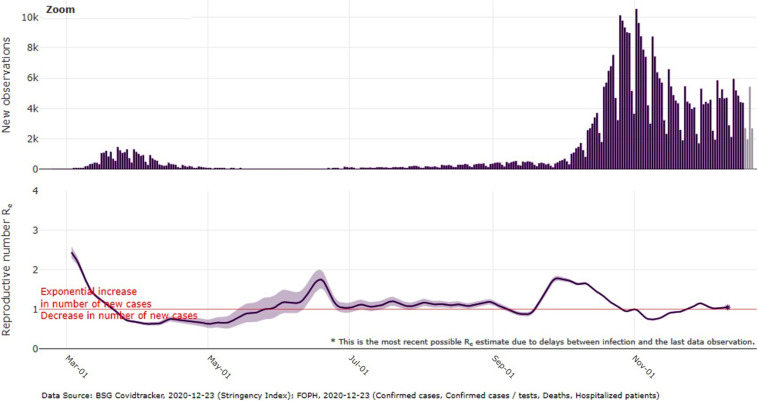
Daily new confirmed COVID-19 cases for Switzerland and effective reproductive number (Re) as estimated and depicted by the Swiss Federal Institute (ETH) Zürich (1st March until 23rd December 2020). Graph retrieved from: https://ibz-shiny.ethz.ch/covid-19-re-international/ (ETH, 23rd December 2020).

Several types of human coronaviruses with low pathogenicity had been studied before the severe acute respiratory syndrome (SARS) emerged in 2002 in China (Drosten et al., 2003; Ksiazek et al., 2003; Peiris et al., 2003). SARS spread to at least 29 countries in Asia, Europe, and North and South America, with a total of 8,098 infections and 774 SARS-related deaths reported (Kahn and McIntosh, 2005). The virus that causes the presently spreading human coronavirus disease, named COVID-19, was first noticed in Wuhan, China, in December 2019, and it resembles the prior SARS (Ali S. A. et al., 2020; Liu et al., 2020; Wu et al., 2020). The infected typically experience symptoms similar to those of a common flu, with an estimated 80% showing only mild symptoms (Hafeez et al., 2020). As of 22nd December 2020, 76,023,488 cases and 1,694,128 deaths have been reported due to COVID-19 worldwide (World Health Organization, 2020a). For Switzerland, there have been 402,264 cases and 5,981 COVID-19-related deaths reported to this date (World Health Organization, 2020b) compared to a resident population of 8.606 million (by the end of 2019, Federal Statistical Office, 2020). The first COVID-19 case in Switzerland was registered on 25th February 2020 (Scire et al., 2020). The first wave of the pandemic took place in late March and early April 2020. By 23rd March, the effective reproductive number (Re)^1^ had decreased below one (95%-confidence interval below one), as depicted in Figure 1, and the first wave was overcome by late May 2020, in the sense that daily new cases had decreased to single digits (Our world data, 2020). Shortly thereafter, the survey was conducted from 16th June until 15th July 2020. The subsequent second wave has recently grown significantly more severe than the first wave, with a maximum 7-day average of 8,064 daily new cases reported on 2nd November 2020, which equals 94 daily cases per 100,000 inhabitants (Swiss Federal Institute ETH, 2020).

The COVID-19 pandemic has induced a global crisis with unusual health-related and economic challenges. It has been claimed to have caused “a significant global shock” (Mishra, 2020) and has even been named “catastrophic” (Maliszewska et al., 2020). As a consequence, the psychological health of individuals and families has been greatly affected, particularly regarding issues such as stress, states of shock, fear, existential anxiety, and grief (Pawar, 2020). Switzerland is no exception. The first wave of the COVID-19 pandemic led to drastic measures by the Swiss federal government, including the mobilization of several thousand Swiss citizens through the militia system of the Swiss army (the greatest mobilization since World War II) (Federal Council, 2020a; Federal Office of Public Health, 2020). The most restrictive phase took place from 16th March until 26th April 2020, which has popularly been referred to in Swiss media as the “lockdown” (Abhari et al., 2020; Neue Zürcher Zeitung, 2020a). Registered unemployment increased from 121,018 to 153,413 people between January and April 2020 (+26.8%, State Secretariat for Economic Affairs, 2020a). After the precautionary measures had been gradually relaxed following 26th April, the Federal Council and the Federal Office of Public Health intensified the measures again in October 2020 in reaction to the second wave (Federal Office of Public Health, 2020). Several branches of the Swiss economy have been under considerable pressure (State Secretariat for Economic Affairs, 2020b), and prognoses for the near future remain unfavorable (State Secretariat for Economic Affairs, 2020c). By the end of November 2020, 153,270 people were registered as unemployed, amounting to an unemployment rate of 3.3% (State Secretariat for Economic Affairs, 2020a). Accordingly, the pressure on the economy is still high, as is the strain on the psychological health of the population, given this ongoing phase of restricted public and private life, economic uncertainty, health hazard, and loss.

Healthcare workers are a primary group on which the COVID-19 pandemic has imposed extraordinary challenges. This has clearly been recognized in the international literature. As first responders in providing care, they have been exposed to feelings of stress and uncertainty, while working long hours and often not being fully protected against an infection (Shaukat et al., 2020). The risk of testing positive for COVID-19 is high among healthcare workers (Nguyen et al., 2020), which, combined with the responsibility they bear for their patients, has exposed them to ethical dilemma (Menon and Padhy, 2020). As private citizens, they have also had to cope with posing an increased infection risk to their social environment. Even being depicted as “heroes” by the media can in fact be counterproductive, as it increases their perceived pressure (Cox, 2020). This situation can significantly affect their mental health and even lead to work-related trauma (Probst et al., 2020; Vagni et al., 2020). Many healthcare workers have been documented to have developed mental issues for which they require psychological support (Lai et al., 2020). This is a clear indication that, besides infrastructural considerations, also the individual capacities of healthcare workers, including their psychological well-being, are a crucial ingredient in facing a pandemic of the magnitude of COVID-19.

Shortly before the first wave of COVID-19 in Switzerland, northern Italy, a direct neighbor, experienced a severe overload of the healthcare system due to COVID-19, particularly of hospitals and intensive care units (ICU). This provided an alarming example to Swiss healthcare workers. The International Council of Nurses (2020) documented both the high rate of infection among healthcare workers in northern Italy, who then needed to be isolated outside of the workforce for 14 days, as well as the physical and mental exhaustion of them and their colleagues who were still/again in service. In mid-October 2020, as the second wave of COVID-19 infections had already emerged, the Swiss Society of Emergency and Rescue Medicine, Switzerland Emergency Care, and the Swiss Association of Paramedics together issued an open call to the Swiss government for support. They stated that the health of Swiss healthcare workers, which had already deteriorated due to the first wave, was at considerable risk of getting worse, if the government did not apply consistent measures across the entire country (SwissInfo.ch, 2020a).

Beyond these challenges, the pandemic has exposed the vulnerability of people, among them also healthcare workers, towards receiving flawed information through popular media, which may affect their judgment. The conveyed information may be imprecise or even misleading, and it may originate within media outlets themselves or merely be transmitted by them. The notion of vast flows of information on a “hot topic” coming from all kinds of sources, of which it may not always be clear to the reader/listener which are proven facts and which are opinions, is known as *infodemics* (Lexico dictionary, 2020). Filtering information by assessing its source is therefore a necessity, particularly for healthcare workers.

With the physical and mental health of healthcare workers being at stake, insight on their perspective and identification of their crucial challenges, as they perceive them, are greatly needed. It is a first step towards sensibly protecting them for their own sake, as well as for them to remain effective and efficient in their services, during a time when they are most needed by society. A rapid and effective response, as well as healthcare staff that is still able to take leadership, are pivotal in successfully handling the pandemic (see e.g., Nagesh and Chakraborty, 2020). Lessons from the first wave of the pandemic are therefore needed, and first-hand empirical data is key. This study presents a quantitative survey of Swiss healthcare workers (*n* = 185) conducted shortly after the first wave of the pandemic. Its aim is to provide evidence of their clinical knowledge about COVID-19, their emotional reaction, their adherence to preventive guidelines, and the impact on their work situation. For such insight to be accurately drawn, understanding the context is essential. Therefore, the circumstances under which the first wave impacted the healthcare workers need to be considered, which to a large degree depend on how the government and the healthcare system were prepared for and reacted to the pandemic.

A few recent studies have provided quantitative evidence of the knowledge of healthcare workers on COVID-19. Wahed et al. (2020) have studied Egyptian healthcare workers, showing that knowledge was higher among the more highly educated individuals, as well as among those below the age of 30 years. Zhang et al. (2020) in their survey of Chinese healthcare workers concluded that knowledge was sufficient in 89% of them. Honarvar et al. (2020) have provided evidence of the knowledge of the general public on certain COVID-19-related issues for the case of Iran. Similarly, Abdelhafiz et al. (2020) have assessed the knowledge of the Egyptian general population. To our knowledge, no study has been published so far specifically focusing on the clinical knowledge of Swiss healthcare workers and their media use. Our study therefore fills in this gap in the literature.

Several studies in the international literature have given insight on personal protective equipment (Park, 2020), specific work risks for healthcare workers related to COVID-19 (Ali S. et al., 2020), and psychological coping mechanisms (see e.g., Muller et al., 2020; Probst et al., 2020; Teo et al., 2020; Vagni et al., 2020). Further studies have shed light on risk perception and attitudes towards COVID-19 (see e.g., Führer et al., 2020; Hager et al., 2020; Honarvar et al., 2020; Zegarra-Valdvia et al., 2020). However, when considering risk perception and attitudes, many of the available studies refer to the general population instead of healthcare workers in particular. Exceptions are given as follows. Spiller et al. (2020), who focused specifically on a sample of Swiss healthcare workers, found no substantial changes in anxiety or depression over the course of the COVID-19 pandemic. Aebischer et al. (2020), who surveyed 227 resident medical doctors and 550 medical students through snowball sampling in Switzerland, found that those medical students who were involved in the COVID-19 response (30%) displayed higher levels of emotional distress than their non-involved peers, and lower levels of burnout compared to the residents. Dratva et al. (2020) analyzed Generalized Anxiety Disorder Scale-7 (GAD-7) in a sample of 2,429 Swiss university students, 595 of which (25%) were students of health professions. They found three classes of individuals regarding the perceived impact of the COVID-19 pandemic, with large differences in the odds of increased anxiety. They concluded that preventive/containment measures against COVID-19 had a selective effect on anxiety in students. However, these analyses were not differentiated across professions/fields, and therefore no results specific to healthcare workers or students of health professions were available. Puci et al. (2020) showed that the risk perception of getting infected with COVID-19 was high among Italian healthcare workers. They also reported sleep disturbances in 64% of the participants, and that 84% perceived a need for psychological support. Abolfotouh et al. (2020) in their survey of Saudi Arabian healthcare workers found that three in four respondents felt at risk of contracting COVID-19 at work, and that 28% did not feel safe at work given the available precautionary measures. Predictors of high concern were, among others, younger age, undergraduate education, and direct contact with patients. In a study of Ethiopian healthcare workers (Girma et al., 2020), risk perception due to the pandemic was measured by ten items on a five-point Likert scale. The mean score of perceived vulnerability was higher for COVID-19 than for the human immunodeficiency virus, the common cold, malaria, and tuberculosis. Wahed et al. (2020) studied a sample of Egyptian healthcare workers, finding that 83% were afraid of being infected with COVID-19. Therein, a lack of protective equipment, fear of transmitting the disease to their families, and social stigma were the most often named reasons. Two further studies are currently in their preprint phase: Firstly, Weilenmann et al. (2020) investigated mental health (depression, anxiety, and burnout) in physicians and nurses from Switzerland, considering work characteristics and demographics as explanatory factors. They concluded that support by the employer, as perceived by the physicians and nurses, was an important indicator of anxiety and burnout, while COVID-19 exposure was not strongly related with mental health. Secondly, Uccella et al. (2020) identified specific risk factors/groups among workers of public hospitals in Italy and Switzerland regarding psychological distress, such as being female and working in intensive care. Having both children and stress symptoms was associated with the perceived need to experience psychological support. Accordingly, while several studies are available regarding specific measures of psychological deterioration, such as anxiety or depression, and also regarding risk perception, quantitative evidence for the specific case of healthcare workers in Switzerland is still rare. Furthermore, the mentioned studies of risk perception referred to the situation at the time of the respective surveys during the pandemic, meaning that the available preventive measures and policies varied substantially. By contrast, the participants of our study were instructed to quantify the risk of COVID-19 independently of the specific precautionary measures that were in place at the time. That is, they answered for the scenario in which no other precautionary measures were taken during the first pandemic wave, other than the usual measures against common influenza. Albeit hypothetical, this allowed for a more general assessment of the threat imposed by COVID-19, making it more comparable to other health hazards.

The precautionary health behavior practices of Ethiopian healthcare workers were assessed by Girma et al. (2020) with a ten-item questionnaire. The items covered dimensions such as the frequency of wearing gloves or wearing a mask. Zhang et al. (2020) surveyed the implementation of four mandatory practices in hospitals among Chinese healthcare workers, concluding that 90% followed them correctly. Our survey contributes to the literature by using a different set of guidelines, which were legally non-binding and issued by the national government towards the general population. Thereby, the study covers the adherence of healthcare workers also in their private life, and is specific to the case of Switzerland.

Several studies have recently examined the responses to the COVID-19 pandemic in different countries. They adopted different perspectives, analyzing the effectiveness of governmental policies (Dergiades et al., 2020; Desson et al., 2020), epidemiological responses (Jefferies et al., 2020), testing, contact tracing and isolation (Salathe et al., 2020), lockdown policy (Faber et al., 2020), preparation of the healthcare sector (Barro et al., 2020), as well as key learned lessons (Han et al., 2020). However, empirical studies of how such measures are perceived by the healthcare staff, and of how the pandemic has affected their work situation from their own perspective, are still scarce. Spiller et al. (2020) compared two demographics-matched samples of healthcare workers, which were collected at two different points in time: at the height of the pandemic (T1) versus two weeks after the healthcare system had started its transition back to usual operations (T2). They found that working hours were higher at T1 compared to T2, and still higher at T2 compared to pre-pandemic levels. Uccella et al. (2020) found that healthcare staff working in intensive care experienced an increase in working hours. The study by Wolf et al. (2020) investigated the effect of policies such as the Swiss “lockdown” on dental practices and social issues such as unemployment and practice closures, assuming on a more economic perspective. Abolfotouh et al. (2020) found broad approval among healthcare workers of the following: the suggestion that the national government in Saudi Arabia should mandate the isolation of COVID-19 patients in specialized hospitals, travel restrictions within the country, and curfew. Our study contributes by providing evidence of how the work situation of healthcare workers had been impacted from their own perspective, and of how they perceived the measures that were implemented by the government.

This study provides insight on several psycho-social factors that in combination are relevant to the role of healthcare workers in the current pandemic. They are not specific psychological diagnoses or concepts of psychological deterioration like depression, anxiety, or burnout, but concern a broader spectrum of issues relevant to the mental wellbeing and the capability to act of healthcare workers. This supports policymakers in pragmatically fostering their comprehensive view of the situation, and in designing policies to sustainably protect the wellbeing of healthcare workers. In addition, the healthcare workers named the specific lessons that needed to be learned from their perspective when facing further pandemic waves.

## Materials and Methods

### Study Setting

This cross-sectional survey was conducted from 16th June to 15th July 2020 with Swiss healthcare workers who regularly worked in direct contact with patients. The healthcare workers were also pursuing a professional development course at Careum Weiterbildung or had attended such a course within recent years. Careum Weiterbildung, situated in Aarau, is one out of several institutions in Switzerland offering extra-occupational courses of professional development (/vocational training) to healthcare workers. These courses vary in duration from 1 day to several days per month over several years and cover a broad range of practice-oriented topics and specializations within healthcare and social sciences. They are often multidisciplinary, and they are aimed at improving care by teaching methods of caregiving, knowledge of practical procedures, communication and organizational skills. Attending such professional development courses is highly common among healthcare workers of all specializations and hierarchical positions in the Swiss healthcare system. Participation was strictly voluntary and anonymous^2^. According to Swiss regulations, no approval by an ethics committee was required for this study.

The participants were surveyed under the following circumstances: After the final day of the above-mentioned “lockdown” during the first wave in Switzerland on 26th April 2020 (see section “Introduction”), the preventive measures had been gradually eased by the national government (Neue Zürcher Zeitung, 2020b; Schweizer Radio und Fernsehen, 2020). From 27th April, businesses offering personal services with physical contact, such as hairdressers, beauty shops, and others, had been allowed to reopen, as well as florists and hardware stores (Federal Council, 2020b). From 11th May, primary and lover secondary school had resumed, and restaurants, markets (also others than food), museums and libraries had been allowed to re-open, along with sport events without physical contact (Federal Council, 2020c). From 28th May, religious events with larger groups of people could be held again (with a protection concept for the participants) (Federal Council, 2020d). From 6th June, private and public events with up to 300 people had been re-allowed, and touristic facilities (such as mountain railway, camping sites, etc.) could re-open. On 15th June, the borders with many countries within the EU/EFTA had been completely re-opened (SwissInfo.ch, 2020b). With the survey starting on 16th June, the participants answered the questionnaire after the first wave of COVID-19 had been overcome, and shortly after the government had relaxed preventive measures to a great extent.

### Participants

All healthcare workers who were part of this study (*n* = 185) were directly attending to patients, with 22% (*n* = 40) of them either working with COVID-19 patients at the time of the survey or being scheduled to work with COVID-19 patients within the following 6 months. One in six individuals (17%, *n* = 31) indicated that because of their health condition, they themselves belonged to a risk group regarding COVID-19. The majority worked in a leading position (56%, *n* = 104) and roughly one in six had a technical lead position (18%, *n* = 33). They came from all major areas of the healthcare system, with 22% (*n* = 40) working in acute care (including psychiatric care), 54% (*n* = 100) in nursing homes, 16% (*n* = 30) in home care, and 12% (*n* = 22) in other areas such as rehabilitation and patient counseling^3^. The median age was 49 years, while the minimum was 23, and the maximum was 68. The vast majority were women (89%, *n* = 164). For further characteristics of the sample, see Table 1.

**TABLE 1:** Demographic and work-related characteristics of healthcare workers in a survey about COVID-19 in Switzerland, June 16th until July 15th 2020 (*n* = 185).

**Works with COVID-19 patients^a^ % (n)**	
Yes	21.6 (40)
No	27.0 (50)
Still undetermined at the time	51.4 (95)
**Health sector (multiple allowed) % (n)**	
Acute care (incl. psychiatric acute care)	21.6 (40)
Nursing homes	54.1 (100)
Home care	16.2 (30)
Other	11.9 (22)
No answer	2.7 (5)
**Specialized field (multiple allowed) % (n)**	
Somatic care	19.5 (36)
Geriatrics	60.0 (111)
Psychiatry	9.2 (17)
Other	22.2 (41)
No answer	2.7 (5)
**Hierarchical level % (n)**	
Leading position	56.2 (104)
Technical lead	17.8 (33)
None of the above	22.7 (42)
No answer	3.2 (6)
**Age (years)**	
Mean ± SD	47.1 ± 9.7
Median (min-max)	49 (23-68)
**Gender, children % (n)**	
Female	88.6 (164)
Has children (of any age)	67.1 (110)
Has children (minors only)	45.7 (75)
Male	11.4 (21)
Has children (of any age)	47.6 (10)
Has children (minors only)	38.1 (8)
**Lives by her-/himself % (n)**	
Yes	15.7 (29)
No	84.3 (156)
**Country^*b*^ % (n)**	
Switzerland	82.7 (153)
Germany	14.1 (26)
Other	3.2 (6)

*^*a*^Within 6 months following the survey. ^*b*^In which most of education has been passed.*

### Data Collection

The data were collected by two-stage cluster sampling, inviting all current and recent attendees (past 8 years) of Careum Weiterbildung for voluntary participation in the survey. A standardized online questionnaire was delivered to 1,747 attendees’ addresses on 16th June via e-mail. 38.1% (*n* = 665) of the delivered messages were opened, and for 36.4% (*n* = 242) thereof the link to the survey was followed, as controlled by *Mailworx* software. A reminder was delivered to 1,684 attendees’ addresses on 30th June, which was opened in 32.9% (*n* = 554) of the cases, and for 29.1% (*n* = 161) thereof the link to the survey was followed. A total of 194 participants completed the questionnaire, 185 of which directly attended to patients and therefore belonged to the population of main interest. Completion took 18.1 min at the median (minimum 9.3; maximum 54.6).

**FIGURE 2:**
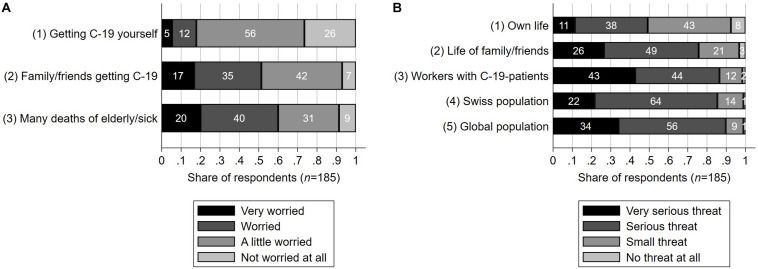
Emotional distress and risk perception of healthcare workers in a survey from Switzerland, June 16th until July 15th 2020 (*n* = 185). **(A)** Emotional distress as measured on a four-point Likert scale in response to the question “how worried do you feel because of the possibility of [the respective scenario]?”. **(B)** Risk perception as measured on a four-point Likert scale. The participants were asked “would COVID-19 be a threat” in the respective regard “if no extraordinary measures were undertaken in Switzerland other than the usual measures against influenza (i.e., no prohibition of social gatherings/events, no lockdown, no extraordinary measures in hospitals)?” **(A,B)** C-19 stands for COVID-19.

**FIGURE 3:**
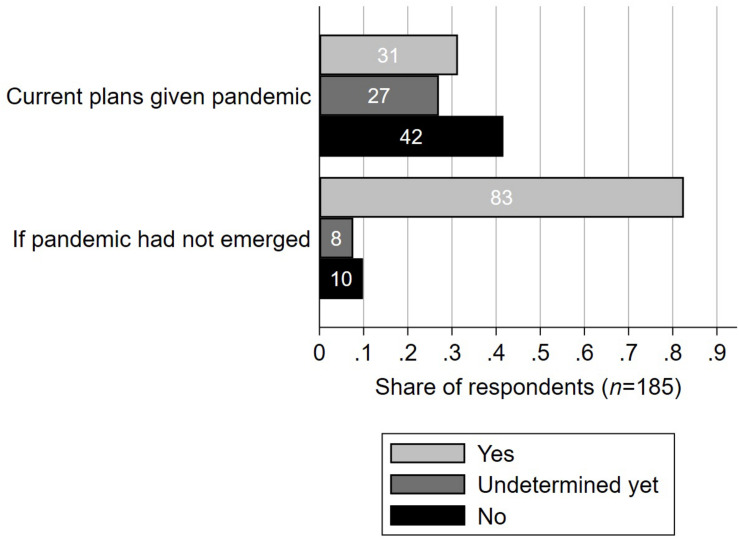
Travel plans given the first wave of the COVID-19 pandemic compared to without the pandemic according to healthcare workers in a survey from Switzerland, June 16th until July 15th 2020 (*n* = 185). Participants were asked “will you travel abroad for private reasons before the end of 2020?” and “would you have traveled abroad for private reasons before the end of 2020 if the COVID-19 pandemic had not occurred?”, respectively.

**FIGURE 4:**
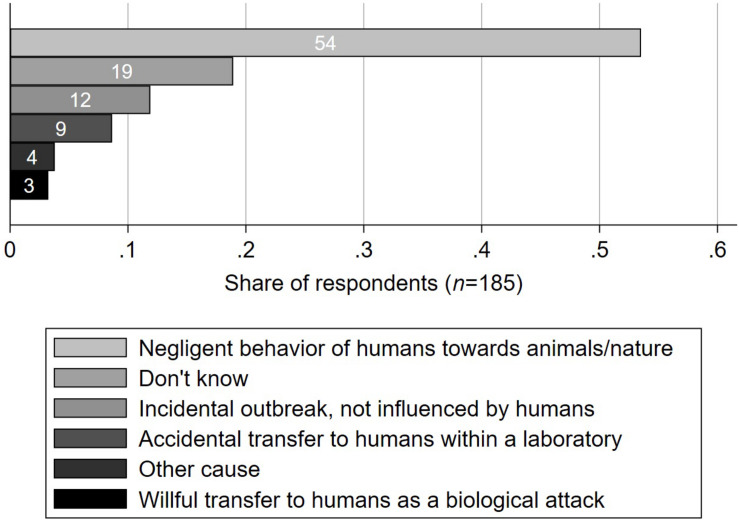
Cause of the outbreak of the COVID-19 pandemic according to healthcare workers in a survey from Switzerland, June 16th until July 15th 2020 (*n* = 185). Participants were asked “what is the cause of the outbreak of the COVID-19 pandemic?”

The questions were posed with given answer options, predominantly in multiple-answer form, and some in multiple-choice form (As the only exception, the participants entered their age as an integer). Thereby, parts of the “Standard questionnaire on risk perception of an infectious disease outbreak” by the Municipal Public Health Service Rotterdam-Rijnmond and the National Institute for Public Health and the Environment (Voeten, 2015) were adapted to the case of the COVID-19 pandemic. The answer option “other” was frequently included which, if selected, led to a request for text input for specification by the participant. Questions were posed across the different parts of the questionnaire as follows. (1) Knowledge about COVID-19: The participants were presented with eight claims about COVID-19 as stated in Table 2 (labeled as items K1-K8). They were asked to choose for each claim whether it was correct, incorrect, or unknown to them (options “right”/“wrong”/“don’t know”). The correct answers shown in Table 2 (“true” or “false” in parenthesis) were taken from the following sources: Day (2020) (K1); Mullard (2020) (K2); Morawska and Cao (2020), World Health Organization (2020c) (K3); Satinder et al. (2020), World Health Organization (2020d), (K4); Osterholm et al. (2020) (K5); NCIRD (2020) (K6); Petersen et al. (2020) (K7); World Health Organization (2020e) (K8). In a second question, they chose from eight different topics (items I1-I8, as listed in Table 2) those on which they needed more detailed information than they had at the time (for the precise wording of the question see Table 2). (2) Sources of information and means of communication: A first multiple-answer question on who should provide them with the necessary information on COVID-19 (seven answer options, S1-S7), as well as a second multiple-answer question on how they preferred to receive this information (ten answer options, M1-M10), measured their preferred media use (see Table 3 for the precise wording). Furthermore, the participants rated their use of each of five given types of media (U1-U5) on a six-point Likert scale ranging from “daily” to “never” (see Table 4 for the precise wording). (3) Emotional distress and risk perception: The first question was “how worried do you feel because of the possibility of [the respective scenario]?” The three scenarios of “getting COVID-19 yourself,” “family/friends getting COVID-19,” and “numerous cases of death among elderly and sick people due to COVID-19” were each rated on a four-point Likert scale ranging from “very worried” to “not worried at all,” as listed in graph A of Figure 2. For the questions on risk perception, a hypothetical scenario was introduced by the wording “please answer for the scenario in which no extraordinary measures were undertaken in Switzerland other than the usual measures against influenza (i.e., no prohibition of social gatherings/events, no lockdown, no extraordinary measures in hospitals).” For this scenario, the question “would COVID-19 be a threat to…” was asked in the five specific respects of “…your own life?”, “…the life of your family members or friends?”, “health professionals attending to COVID-19 patients?”, “…the Swiss population?”, and “…the global population?”. The answers were given on a four-point Likert scale ranging from “very serious threat” to “no threat at all,” as listed in graph B of Figure 2. As a follow-up, the identical questions were asked a second time, with the answers on a discrete rating scale as described by Studer and Winkelmann (2017). The discrete rating scale ranged from zero to ten, and only the extremes were verbally labeled (“0 = no threat at all;” “10 = very serious threat”). This allowed for the application of different methods of analysis, as described in the section “Data Analysis.” (4) Perception of and adherence to preventive guidelines: The participants rated the likelihood of a second wave of COVID-19 in Switzerland before the end of 2020 on a six-point Likert scale ranging from “certainly” to “certainly not.” They also rated the likelihood of a different pathogen causing another pandemic of equivalent or greater magnitude within the upcoming 20 years on the same scale. Table 5 lists the precise wording of the question and the answer options. Note that for the intermediate levels of the Likert scale, the resulting frequencies are presented in cumulative form, as described in the section “Results.” In the questionnaire, the Likert scale was included in typical fashion without cumulative meaning (i.e., no “≥” or “≤” signs). The participants repeated the assessment of the same two questions, but this second time with the answer options being on a discrete rating scale ranging from one to ten with only the extremes having a verbal label (“0 = certainly not;” “10 = certainly”). They were then shown six preventive guidelines (A1 and A3-A7 in Table 6). These guidelines were in place in Switzerland during the “lockdown” phase (with A3 and A4 formulated slightly less strictly/clearly), and some of them were relaxed afterwards. However, they had the status of recommendations by the federal government, not of legally binding rules. The participants indicated how strictly they followed them on a six-point Likert scale ranging from “always” to “never.” The precise wording is given in Table 6. Like in Table 5, while the resulting frequencies for the intermediate levels are presented in their cumulative form, this was not the case in the questionnaire, where the ordinary Likert scale was used (without “≥” or “≤” signs). The participants were further asked to indicate how strictly they expected to follow the same guidelines in the future, as listed in the lower part of Table 6 (A11 and A13-A17). There, the six-point Likert scale ranged from “presumedly forever” to “0 to 1 month,” and the alternative option of “don’t know” was added. To evaluate these guidelines, the participants were asked “which of the following claims apply to the above-mentioned guidelines?” referring to guidelines A1 and A3 through A7. They were presented with the multiple answer options “most of them are exaggerated for persons *not* working with patients or elderly people,” “most of them are exaggerated for persons working with patients or elderly people,” “most of them are ineffective,” and “none of the answers above apply.” Finally, the participants indicated whether they currently had any plans of traveling abroad for private reasons before the end of the year 2020 (multiple-choice options “yes”/“no”/“undetermined yet”), and whether they would have had such plans if the COVID-19 pandemic had not occurred (see the precise wording in Figure 3). (5) impact on work situation: For each of four claims regarding preparation (P1-P4 as shown in Table 7) it was asked whether the claim was true or not. By item P5 the choice was offered that none of the claims P1 through P4 were true, which, if chosen, implied that P1 through P4 could not be selected as well. The question “how has/had COVID-19 affected your work situation?” was then asked with eleven answer options (W1-W11 as listed in Table 7) of which the last option excluded all other ten. (6) Reaction by the government: The sentence “the measures implemented by the government between 17th March and 26th April (“lockdown”) were…” could be completed with either “…exaggerated,” “…adequate,” or “…not strict enough / too late / too short in duration.” The follow-up question was “which of the following claims applies to the gradual steps of relaxation of these measures, which are in place since 27th April and which are planned for the future?”. The multiple-choice answer options were “the measures should have been relaxed earlier / more strongly,” “the relaxation plan is adequate,” and “the measures should have been relaxed later / less strongly.” (7) Key lessons: The question “which lessons need to be learned and what should be different in case another pandemic should happen in the future?” was asked with ten answer options (L1-L10 as listed in Table 7) of which the last one excluded all other options. (8) Presumed cause of the pandemic: The participants were presented with a multiple-choice question phrased as shown in Figure 4. At the end of the questionnaire, the participants could enter any comments, regardless of their previous answers.

**TABLE 2:** Knowledge of healthcare workers regarding COVID-19 and their needs for information in a survey from Switzerland, June 16th until July 15th 2020 (*n* = 185).

**No.**	**Item**	**Freq.**	**CI (Wilson)**	
		**% (n)**	**%**	**%**
**Correct indication provided on the following statements being true/false.**				
K1	COVID-19 leads to symptoms in every case. (False)	92.4 (171)	87.7	95.4
K2	There currently (June/July 2020) is an effective vaccination against COVID-19. (False)	95.1 (176)	91.0	97.4
K3	COVID-19 is transmitted between people exclusively via physical contact. (False)	91.9 (170)	87.1	95.0
K4	If hygiene standards such as frequent washing of hands and sneezing only into tissues are met, an infection with COVID-19 is virtually impossible. (False)	57.3 (106)	50.1	64.2
K5	COVID-19 has a higher infectiousness than influenza. (True)	75.7 (140)	69.0	81.3
K6	COVID-19 has a shorter incubation time than influenza. (False)	72.4 (134)	65.6	78.4
K7	COVID-19 has a higher rate of life-threatening disease progression than influenza. (True)	68.6 (127)	61.6	74.9
K8	Vaccines against influenza are also effective against COVID-19. (False)	93.5 (173)	89.0	96.3

**Question: On which COVID-19-related topics do you need more detailed information than you presently have?**				
I1	Transmission between people.	14.6 (27)	10.2	20.4
I2	Incubation time.	33.5 (62)	27.1	40.6
I3	Symptoms.	10.8 (20)	7.1	16.1
I4	Preventive measures.	13.0 (24)	8.9	18.6
I5	Infectiousness.	26.5 (49)	20.7	33.3
I6	Severe disease progression.	29.2 (54)	23.1	36.1
I7	Treatment.	42.7 (79)	35.8	49.9
I8	Other.	8.1 (15)	5.0	12.9

**TABLE 3:** Preferences of healthcare workers on sources of information and means of communication in a survey from Switzerland, June 16th until July 15th 2020 (*n* = 185).

**No.**	**Item**	**Freq.**	**CI (Wilson)**	
		**% (n)**	**%**	**%**
**Question: Who should provide you with the necessary information on COVID-19?**				
S1	Employer.	60.5 (112)	53.4	67.3
S2	General practitioner.	26.5 (49)	20.7	33.3
S3	Hospitals.	14.6 (27)	10.2	20.4
S4	Government (municipal, cantonal, federal).	81.1 (150)	74.8	86.1
S5	Journalists/publishers.	11.9 (22)	8.0	17.3
S6	Scientists/universities.	62.7 (116)	55.5	69.3
S7	Other.	3.2 (6)	1.5	6.9

**Question: How do you prefer to receive the necessary information on COVID-19?**				
M1	Postal delivery.	18.4 (34)	13.5	24.6
M2	Billboards.	28.6 (53)	22.6	35.5
M3	Public television.	74.6 (138)	67.9	80.3
M4	Advertisements in newspapers.	9.2 (17)	5.8	14.2
M5	Newspaper articles.	56.8 (105)	49.6	63.7
M6	Radio.	65.9 (122)	58.9	72.4
M7	Leaflets.	6.5 (12)	3.7	11.0
M8	Orally by employer.	16.2 (30)	11.6	22.2
M9	In writing by employer.	56.2 (104)	49.0	63.2
M10	Other.	5.9 (11)	3.4	10.3

**TABLE 4:** Regular media use of healthcare workers in a survey from Switzerland, June 16th until July 15th 2020 (*n* = 185).

**No**	**Item**	**% (n)**	**% (n)**	**% (n)**	**% (n)**	**% (n)**	**% (n)**
		**[CI %]**	**[CI %]**	**[CI %]**	**[CI %]**	**[CI %]**	**[CI %]**
**Question: How often do you usually (not only regarding COVID-19) use the following media to keep informed on recent news?**		**Daily**	**≥ Several times a week**	**≥ Once a week**	**≥ Once a month**	**≥ Less than once a month^*a*^**	**Never**
U1	Daily newspapers requiring subscription (also digital).	38.4 (71) [31.7; 45.6]	53.5 (99) [46.3; 60.6]	61.1 (113) [53.9; 67.8]	65.4 (121) [58.3; 71.9]	68.1 (126) [61.1; 74.4]	31.9 (59) [25.6; 38.9]
U2	Free daily newspapers without subscription (also digital).	33.5 (62) [27.1; 40.6]	56.2 (104) [49.0; 63.2]	73.0 (135) [66.2; 78.9]	79.5 (147) [73.1; 84.7]	88.1 (163) [82.7; 92.0]	11.9 (22) [8.0; 17.3]
U3	TV programs (also via internet).	36.2 (67) [29.6; 43.4]	71.9 (133) [65.0; 77.9]	88.1 (163) [82.7; 92.0]	93.5 (173) [89.0; 96.3]	96.8 (179) [93.1; 98.5]	3.2 (6) [1.5; 6.9]
U4	Radio programs (also via internet).	41.1 (76) [34.2; 48.3]	71.9 (133) [65.0; 77.9]	82.7 (153) [76.6; 87.5]	87.0 (161) [81.4; 91.1]	93.0 (172) [88.3; 95.8]	7.0 (13) [4.2; 11.7]
U5	News automatically suggested by Google or other web browsers.	13.5 (25) [9.3; 19.2]	38.9 (72) [32.2; 46.1]	51.4 (95) [44.2; 58.5]	60.0 (111) [52.8; 66.8]	73.5 (136) [66.7; 79.3]	26.5 (49) [20.7; 33.3]

*^*a*^“Less than once a month” excluded “never.” “≥Less than once a month” encompasses all individuals who answered “less than once a month,” “once a month,” “once a week,” “several times a week” or “daily.” “CI” stands for Wilson’s confidence interval.*

**TABLE 5:** Likelihood of further pandemic waves after the first wave of COVID-19 according to healthcare workers in a survey from Switzerland, June 16th until July 15th 2020 (*n* = 185).

**No**	**Item**	**% (n)**	**% (n)**	**% (n)**	**% (n)**	**% (n)**	**% (n)**
		**[CI %]**	**[CI %]**	**[CI %]**	**[CI %]**	**[CI %]**	**[CI %]**
**Question: How likely is the following to take place?**		**Certainly**	**≥Very likely**	**≥ Rather likely**	**≤Rather unlikely**	**≤ Very unlikely**	**Certainly not**
F1	A second wave of COVID-19 infections in Switzerland beginning before the end of the year 2020.	11.4 (21) [7.5; 16.7]	36.8 (68) [30.1; 43.9]	77.8 (144) [71.3; 83.2]	22.2 (41) [16.8; 28.7]	2.2 (4) [0.8; 5.4]	0.5 (1) [0.1; 3.0]
F2	A different pathogen causing another pandemic of equivalent or greater magnitude than COVID-19 within the next 20 years.	12.4 (23) [8.4; 18.0]	43.2 (80) [36.3; 50.4]	88.6 (164) [83.3; 92.5]	11.4 (21) [7.5; 16.7]	2.7 (5) [1.2; 6.2]	0.5 (1) [0.1; 3.0]

*The six answer options were *“certainly,” “very likely,” “rather likely,” “rather unlikely,” “very unlikely,” and “certainly not*;*”* “*≥Rather likely”* encompasses all individuals who answered “*rather likely*,” “*very likely*,” or “*certainly*;” “*≤Rather unlikely”* encompasses all individuals who answered “*rather unlikely*,” “*very unlikely*,” or “*certainly not*.” “CI” stands for Wilson’s confidence interval.*

**TABLE 6:** Adherence to preventive guidelines of healthcare workers after the first wave of COVID-19 in a survey from Switzerland, June 16th until July 15th 2020 (*n* = 185).

**No**	**Item**	**% (n)**	**% (n)**	**% (n)**	**% (n)**	**% (n)**	**% (n)**	**% (n)**
		**[CI %]**	**[CI %]**	**[CI %]**	**[CI %]**	**[CI %]**	**[CI %]**	**[CI %]**
**Question: How strictly do you follow these guidelines?**		**Always**	**≥Almost always**	**≥ Pre-dominantly**	**≤Some-times**	**≤ Almost never**	**Never**	**-^*b*^**
A1	Make no use of public transportation during rush hour.	55.7 (103) [48.5; 62.6]	74.6 (138) [67.9; 80.3]	82.2 (152) [76.0; 87.0]	17.8 (33) [13.0; 24.0]	10.3 (19) [6.7; 15.5]	8.1 (15) [5.0; 12.9]	-
A3^*a*^	Keep a physical distance of at least two meters from everyone except your closest family.	8.1 (15) [5.0; 12.9]	50.3 (93) [43.1; 57.4]	80.5 (149) [74.2; 85.6]	19.5 (36) [14.4; 25.8]	5.4 (10) [3.0; 6.7]	0.5 (1) [0.1; 3.0]	-
A4	Disinfect or wash your hands with soap for 20 seconds after each physical contact, except with family.	35.7 (66) [29.1; 42.8]	66.5 (123) [59.4; 72.9]	89.2 (165) [83.9; 92.9]	10.8 (20) [7.1; 16.1]	3.8 (7) [1.8; 7.6]	0 (0) [0; 2.0]	-
A5	Do not shake hands.	82.2 (152) [76.0; 87.0]	95.7 (177) [91.7; 97.8]	97.3 (180) [93.8; 98.8]	2.7 (5) [1.2; 6.2]	1.6 (3) [0.6; 4.7]	0.5 (1) [0.1; 3.0]	-
A6	Cough and sneeze only into a tissue or the inside of your elbow if no tissue is available.	89.2 (165) [83.9; 92.9]	96.8 (179) [93.1; 98.5]	98.9 (183) [96.1; 99.7]	1.1 (2) [0.3; 3.9]	1.1 (2) [0.3; 3.9]	0 (0) [0; 2.0]	-
A7	In case of a cough or fever, do not leave your home and contact the hotline or a physician via phone.	80.5 (149) [74.2; 85.6]	89.2 (165) [83.9; 92.9]	91.9 (170) [87.1; 95.0]	8.1 (15) [5.0; 12.9]	3.2 (6) [1.5; 6.9]	2.7 (5) [1.2; 6.2]	-

**Question: How strictly do you expect to follow these guidelines in the future with the same intensity as you indicated above?**		**Presumedly forever**	** ≥ Until vaccine available**	**≥ 7 to 12 months**	** ≥ 4 to 6 months**	**≥ 2 to 3 months**	**0 to 1 month**	**Don’t know**
A11	Make no use of public transportation during rush hour.	16.2 (30) [11.6; 22.2]	34.6 (64) [28.1; 41.7]	44.3 (82) [37.4; 51.5]	55.1 (102) [47.9; 62.1]	63.2 (117) [56.1; 69.9]	8.1 (15) [5.0; 12.9]	28.6 (53) [22.6; 35.5]
A13^*a*^	Keep a physical distance of at least two meters from everyone except your closest family.	7.6 (14) [4.6; 12.3]	30.8 (57) [24.6; 37.8]	38.4 (71) [31.7; 45.6]	50.8 (94) [43.7; 57.9]	61.6 (114) [54.4; 68.3]	14.6 (27) [10.2; 20.4]	23.8 (44) [18.2; 30.4]
A14	Disinfect or wash your hands with soap for 20 seconds after each physical contact, except with family.	34.6 (64) [28.1; 41.7]	54.6 (101) [47.4; 61.6]	63.8 (118) [56.6; 70.4]	71.9 (133) [65.0; 77.9]	80.0 (148) [73.7; 85.1]	5.4 (10) [3.0; 9.7]	14.6 (27) [10.2; 20.4]
A15	Do not shake hands.	23.8 (44) [18.2; 30.4]	44.9 (83) [37.9; 52.1]	54.6 (101) [47.4; 61.6]	63.2 (117) [56.1; 69.9]	70.8 (131) [63.9; 76.9]	7.0 (13) [4.2; 11.7]	22.2 (41) [16.8; 28.7]
A16	Cough and sneeze only into a tissue or the inside of your elbow if no tissue is available.	85.4 (158) [79.6; 89.8]	91.9 (170) [87.1; 95.0]	93.0 (172) [88.3; 95.8]	95.1 (176) [91.0; 97.4]	95.1 (176) [91.0; 97.4]	1.1 (2) [0.3; 3.9]	3.8 (7) [1.8; 7.6]
A17	In case of a cough or fever, do not leave your home and contact the hotline or a physician via phone.	26.5 (49) [20.7; 33.3]	46.5 (86) [39.4; 53.7]	58.9 (109) [51.7; 65.8]	67.0 (124) [60.0; 73.4]	73.0 (135) [66.2; 78.9]	6.5 (12) [3.7; 11.0]	20.5 (38) [15.3; 26.9]

*^*a*^Items A2 and A12 of the questionnaire were not included in this survey. ^*b*^“Don’t know” was not given as a response option for items A1-A7. For items A1-A7, the six answer options were *“always,” “almost always,” “predominantly,” “sometimes,” “almost never,” and “never*;*”* “*≥Predominantly”* encompasses all individuals who answered “*predominantly*,” “*almost always*,” or “*always*;” for items A11-A17, the seven answer options were *“presumedly forever,” “until vaccine available,” “7 to 12 months,” “4 to 6 months,” “2 to 3 months,” “0 to 1 month*;*”* “*≥Until vaccine available”* encompasses all individuals who answered *“until vaccine available”* or *“presumedly forever*;*” “≥2 to 3 months”* encompasses all individuals who answered *“2 to 3 months, ““4 to 6 months,” “7 to 12 months,” “until vaccine available,”* or *“presumedly forever*;*” “0 to 1 month”* encompasses all individuals who answered *“0 to 1 month*;*”* “CI” stands for Wilson’s confidence interval.*

**TABLE 7:** Assessment of the preparation for a viral pandemic, the work situation due to COVID-19, and the lessons to be learned from the first wave of COVID-19, according to healthcare workers in a survey from Switzerland, June 16th until July 15th 2020 (*n* = 185).

**No**	**Item**	**Freq.**	**CI (Wilson)**	
		**% (n)**	**%**	**%**
**Question: Which of the following claims are true? Ahead of the outbreak of COVID-19, the government and the healthcare system were sufficiently prepared for a viral pandemic with…**				
P1	Disinfectant and protective masks.	9.2 (17)	5.8	14.2
P2	Personnel.	13.5 (25)	9.3	19.2
P3	Structures.	22.7 (42)	17.3	29.3
P4	Processes and contingency plans.	30.3 (56)	24.1	37.2
P5	None of the above claims are true. In none of these four areas were the government and the healthcare sector sufficiently prepared.	58.4 (108)	51.2	65.2

**Question: How has/had COVID-19 affected your work situation?**				
W1	I feel more stressed than usual.	44.3 (82)	37.4	51.5
W2	I have to work more than usual.	32.4 (60)	26.1	39.5
W3	I am more often pressed for time than usual.	17.8 (33)	13.0	24.0
W4	I have to do tasks which are unusual to me.	37.8 (70)	31.2	45.0
W5	I work for a department/division (at least in part) which I do not usually work for.	8.1 (15)	5.0	12.9
W6	My employer shows less consideration for my needs than usual.	18.4 (34)	13.5	24.6
W7	The material and structures necessary to effectively protect the staff from an infection with COVID-19 are available.	71.9 (133)	65.0	77.9
W8	The decisions necessary to effectively protect the staff from an infection with COVID-19 are taken.	81.1 (150)	74.8	86.1
W9	A relevant share of nurses does not strictly abide to the hospital-/institution-specific regulations regarding protective masks, washing of hands, and physical distancing.	22.7 (42)	17.3	29.3
W10	Other.	9.7 (18)	6.2	14.9
W11	Not at all.	0.5 (1)	0.1	3.0

**Question: Which lessons need to be learned and what should be different in case another pandemic should happen in the future?**				
L1	Earlier warning.	31.9 (59)	25.6	38.9
L2	More personnel available/assigned.	36.8 (68)	30.1	43.9
L3	More detailed/accurate information about the symptoms caused by the virus.	35.7 (66)	29.1	42.8
L4	More/better medical equipment (including drugs).	57.8 (107)	50.6	64.7
L5	Keep work schedules as usual (“business as usual”).	13.5 (25)	9.3	19.2
L6	Increased hourly wages due to the exceptional circumstances.	36.8 (68)	30.1	43.9
L7	Better protection for own physical health.	40.0 (74)	33.2	47.2
L8	Better protection for own mental health.	43.8 (81)	36.8	51.0
L9	Other.	13.5 (25)	9.3	19.2
L10	No lessons to be learned or changes needed, as preparation and handling of COVID-19 was appropriate.	6.5 (12)	3.7	11.0

### Data Analysis

Confidence intervals (CIs) of proportions, as shown in Table 2 through Table 7, as well as referred to in the text of the “Results” section, were calculated by Wilson’s method (for a comparison of methods, see Newcombe, 1998). Fisher’s exact test was used for testing the equality of proportions (see section “Emotional Distress and Risk Perception”). Pair-wise rank correlation was calculated by Spearman’s method (see Table 8) and classified according to Cohen (1992). For any tests of hypotheses, whether univariate or within a multiple regression model, a type-one error probability (*p*) < 0.05 was considered as “statistically significant.” In the same regard, alternative hypotheses were two-sided. By binary logistic regression, the effects of multiple predictors on a binary outcome were modeled. The results were computed as average marginal effects (AME) representing percentage-point differences in the probability of the outcome being positive. By fractional logistic rating scale regression, the effects of multiple predictors on an outcome on an eleven-point discrete numeric rating scale (0-10, with labeled extremes) were modeled. The results were represented as AME representing differences on the 0-10 scale. For an explanation of this method, see e.g., Studer and Winkelmann (2017). Each regression model was optimized such that systematic factor elimination minimized Bayes’ information criterion (BIC)^4^. The following models were estimated for the different parts of the questionnaire. (1) Knowledge about COVID-19: A binary logistic model of item K4 (Table 2) being answered correctly (versus wrongly or by the answer option “don’t know”). (3) Emotional distress and risk perception: Three binary logistic models, one for each of the three dimensions depicted in graph A in Figure 2, of the respective outcome being at least “worried” (i.e., (“worried” or “very worried”) versus (“a little worried” or “not worried at all”)). A fractional logistic model of the perceived threat to one’s own life on the 0-10 discrete rating scale, as well as another fractional logistic model of the perceived threat to the life of family members and friends on the same scale. (4) Perception of and adherence to preventive guidelines: Three binary logistic models, one each for the items A1, A3, and A4 (Table 6), of the respective outcome being at least “almost always” (i.e., “almost always” or “always” versus all other answer options). Three binary logistic models, one each for the items A13, A14, and A15 (Table 6), conducted for those participants who claimed to adhere to the respective guideline at least “predominantly” at the time of the survey (as measured by items A3, A4, and A5). Thereby, the probability of continuing the individual level of adherence at least until a vaccine would be available was modeled (i.e., “until vaccine available” or “presumably forever” versus all other answer options, except for “don’t know” in which case the respective individual was excluded). A binary logistic model of currently having plans of traveling abroad before the end of 2020 given the pandemic, as described in Figure 3 (i.e., “yes” versus the other two answer options). (6) Reaction by the government: A binary logistic model of the question “which of the following claims applies to the gradual steps of relaxation of these measures, which are in place since 27th April and which are planned for the future?” being answered by “the measures should have been relaxed later/less strongly” (versus the other two answer options). For each of these BIC-optimized models, all of the predictors and their estimated effects are reported in the “Results” section.

**TABLE 8:** Pairwise rank correlation among items of present adherence to preventive guidelines (*n* = 185) and among items of expected future adherence to preventive guidelines (*n* = 95) according to healthcare workers after the first wave of COVID-19 in a survey from Switzerland, June 16th until July 15th 2020.

**Item-No**	**A1**	**A3^a^**	**A4**	**A5**	**A6**
*Item-No*	*A11*	*A13*^*a*^	*A14*	*A15*	*A16*
A3^a^ *A13*^*a*^	0.160* 0.696***				
A4 *A14*	0.128 0.552***	0.502*** 0.707***			
A5 *A15*	0.126 0.585***	0.402*** 0.662***	0.306*** 0.760***		
A6 *A16*	0.005 0.376***	0.218** 0.341***	0.226** 0.465***	0.265*** 0.392***	
A7 *A17*	0.068 0.702***	0.207** 0.603***	0.230** 0.614***	0.201** 0.585***	0.188* 0.412***

^*a*^Items A2 and A12 of the questionnaire were not used in this survey. Top row within each cell shows pairwise correlation of items A1-A7, referring to present adherence at the time of the survey (June 16th until July 15th 2020); Bottom row within each cell (italics) shows pairwise correlation of items *A11*-*A17*, referring to expected duration of adherence (for the *n* = 95 individuals who did not answer *“don’t know”*); The meaning of the items is listed in Table 6. *p < 0.05, **p < 0.01, ***p < 0.001.

One of the tested predictors in the above-mentioned models concerned a specific public announcement by the Swiss Federal Council, which requires specific explanation. It was made shortly after the start of the survey: During the day of 19th June 2020, the Federal Council announced that most of the national preventive measures in place at that time would be abolished or relaxed on June 22nd. In particular, organized events with up to 1,000 people would be legalized again, the recommended physical distance between people would be reduced from 2 to 1.5 meters, masks would not be mandatory in public transportation (yet recommended), and home office would no longer be a recommendation (Federal Council, 2020e). The Federal Council further announced that the handling of a potential second wave would be the duty of the Swiss cantons, which are the member states of the Swiss Federation. It thereby undertook a fundamental change of policy, which it underlined by suspending the national coronavirus task force (KSBC). Notably, these steps were not known to the broad public before 19th June. Hence, the government’s future plans changed on the 19th of June to being significantly more liberal than before, as far as public knowledge is concerned. From 16th June until 19th June, 107 of the total of 185 participants had already answered the survey. Naturally, by the time the survey had started on 16th June, no question specifically referring to the announcement of 19th June could have been included in the questionnaire. For reasons of consistency, the questionnaire was not altered after the start. Therefore, the day of participation in the survey (i.e., whether it was after 19th June or not) was used as a predictor of the answer to whether the participants agreed with the steps of relaxation “undertaken since 27th April and planned for the future” (see section “Reaction by the Government”).

## Results

### Knowledge About COVID-19

Knowledge was high regarding the unavailability of a COVID-19 vaccine (item K2), the ineffectiveness of influenza vaccines against COVID-19 (K8), the occurrence of symptoms (K1), and transmission without physical contact (K3), with over 92% (confidence intervals (CIs) over 87%) answering correctly (see Table 2). 76% of the participants answered correctly that COVID-19 was more infectious (K5) and 72% that it had a longer incubation time (K6) than common influenza. 69% correctly indicated that COVID-19 cases more often had a life-threatening disease progression than common influenza (K7). However, 36% (CI 29-43%) falsely believed that if hygiene standards such as frequent washing of hands and sneezing only into tissues were met, an infection with COVID-19 would be virtually impossible. Another 7% (CI 4-12%) answered that they did not know the answer to this question. Hence, knowledge on the latter item (K4) was significantly lower than on any other tested item. It was even *lower* among participants who as a result of the pandemic worked more hours than usual (AME = –17.7 percentage points, *p* < 0.05, binary logistic regression).

Additional information on treatment was most frequently desired (43%, I7 in Table 2), followed by incubation time (34%, I2), severe disease progression (29%, I6), infectiousness (27%, I5), transmission between people (15%, I1), preventive measures (13%, I4), and symptoms (11%, I3). 28% (CI 22-35%) claimed not to be needing any further information on COVID-19-related topics (i.e., none of the items I1 through I8 were selected).

Even though knowledge was comparably low regarding the effectiveness of standard hygiene (K4), the topics of preventive measures (I4) and transmission (I1) were rarely named as topics for which further information was perceived to be needed. In fact, among those participants who did not provide the correct answer to this item (K4) (*n* = 79), 85% (CI 75-91%) claimed to be needing no further information on preventive measures (I4), and 86% (CI 77-92%) claimed to be needing no further information on transmission between people (I1). Similar results were found for other topics: Of the participants who did not answer correctly on life-threatening disease progression (K7) (*n* = 58), 74% (CI 62-84%) claimed to be needing no further information on the topic (I6). Of the participants who did not answer correctly on incubation time (K6) (*n* = 51), 45% (CI 32-59%) claimed to be needing no further information on the topic (I2). Of the participants who did not answer correctly on infectiousness (K5) (*n* = 45), 73% (CI 59-84%) claimed to be needing no further information on the topic (I5). This is clear evidence that, although knowledge was fairly high on some topics, many participants overestimated their knowledge (or for other reasons thought that no further information was needed).

### Sources of Information and Means of Communication

The vast majority of the participants (81%) expected the government to be their source of necessary information on COVID-19, as shown in Table 3 (S4), while 63% (also) wished for scientists/universities (S6), and 61% (also) wished for their employer to take on that role (S1). Any other sources were significantly less often named. The most preferred means of communication by which to receive the information were public television (75%, M3), radio (66%, M6), and newspaper articles (57%, M5). Of those participants who wished to receive the information by their employer (*n* = 112, S1), 93% (CI 87-96%) required to receive it in writing (M9), and only 27% (19-36%) orally (also) (M8). Accordingly, television (72%, U3) and radio (72%, U4) were the most popular media in order to keep informed (“several times a week” or “daily”) on recent news in general, not only related to COVID-19 (see Table 4). Still, more than half of the participants read articles by daily newspapers at least “several times a week” (54% for newspapers requiring subscription, U1; 56% for free newspapers, U2). News automatically suggested by web browsers (U5) were significantly less popular than the other mentioned media.

### Emotional Distress and Risk Perception

Merely 18% (CI 13-24%) of the participants felt at least worried (i.e., “worried” or “very worried”) about getting infected with COVID-19 themselves (see graph A in Figure 2). By contrast, 52% (CI 44-58%) felt at least worried about possibly the same happening to their family/friends. 60% (CI 53-68%) felt at least worried about the possibility of numerous deaths among elderly or sick people (people not necessarily personally known to them). Hence, the participants were significantly more often at least worried (i.e., “worried” or “very worried”) about other people being at risk than about themselves (*p* < 0.001, for both bivariate comparisons, Fisher’s exact test). Participants working in long-term care were *more* likely to feel at least worried (i.e., “worried” or “very worried”) about contracting COVID-19 themselves (AME = 0.335, *p* < 0.05, binary logistic regression), participants who had passed the majority of their education in Germany were *more* likely to feel at least worried about their family/friends contracting it (AME = 0.263, *p* < 0.01), and both participants working in somatic care (AME = 0.258, *p* < 0.001) and participants working in nursing homes (AME = 0.284, *p* < 0.001) were *more* likely to feel at least worried about deaths among elderly or sick people.

The provided answers on how severe of a threat COVID-19 was for specific groups are illustrated by graph B in Figure 2. This pertains to the hypothetical scenario without precautionary measures because of COVID-19 other than the usual ones against a common flu (“business as usual”). 90% (CI 85-93%) claimed an at least serious (i.e., “serious” or “very serious”) threat for the global population, and 86% (CI 81-91%) claimed so for healthcare workers who directly attended to COVID-19 patients. 85% (CI 80-90%) claimed an at least serious threat for the Swiss population, and 76% (CI 69-81%) claimed so for the life of their family members and friends. Only 49% (CI 42-56%) claimed an at least serious threat for the global population. Again, a pattern showed according to which the participants significantly more often saw other groups than themselves as threatened (*p* < 0.001, for all four bivariate comparisons, Fisher’s exact test), which is analogous to the observed pattern of emotional distress. The results of the assessment on the discrete 0-10 rating scale were consistent with those on the Likert scale. The proportion of participants who estimated a *strictly lower threat* of COVID-19 *to their own life* was 65% (CI 59-73%) compared to the global population, 64% (CI 56-71%) compared to healthcare workers directly attending to COVID-19 patients, 57% (CI 50-65%) compared to the Swiss population, and 51% (CI 44-59%) compared to their own family and friends. Vice versa, the proportion of participants who estimated a higher threat to their own life than to another group was a single-digit percentage (for any of the four comparisons). Furthermore, 38% (CI 31-46%) claimed that there was a greater threat to the global population than to the Swiss population, and only 4% (CI 2-8%) claimed vice versa. The observation that healthcare workers who directly attended to COVID-19 patients were predominantly estimated to be more threatened than one’s own life calls for closer consideration. It applied even among those participants who themselves attended to COVID-19 patients (n = 40, therein: 58% with CI 41–73%; vice versa 3% with CI 0–13%). This is remarkable, as the majority therein claimed a lower threat for themselves individually than for others, even though they belonged to the very group they were comparing themselves to. While this may appear somewhat paradoxical at first glance, it is another occurrence of the above-mentioned pattern, this time within the group of their peers. Participants who themselves were part of a risk group regarding COVID-19 because of their health condition estimated the threat to their own life to be *higher* (AME = 2.43 points, *p* < 0.001, with a mean outcome over all individuals of 5.47 points on the 0-10 scale), which is unsurprising (as derived by the fractional logistic regression model). The same participants also estimated the threat to the life of their family members and friends to be *higher* (AME = 1.31 points, *p* < 0.001, with a mean outcome over all individuals of 6.80 on the 0-10 scale).

### Perception of and Adherence to Preventive Guidelines

Table 5 tabulates the cumulative distribution of the perceived likelihood of a second wave of COVID-19 and of another pandemic in the future. Note that this is the cumulative distribution over the Likert scale, which is split in its middle such that the left side of the table cumulates frequencies from high to low likelihoods, starting on the left with the highest (“certainly”), and the right side of the table cumulates frequencies from low to high likelihoods, starting from the right with the lowest (“certainly not”). 78% (CI 71-83%, F1) estimated a second wave of COVID-19 to be at least rather likely (i.e., “rather likely,” “very likely,” or “certain”), and 89% (CI 83-93%, F2) estimated such a likelihood of another pandemic in the future. On the discrete 0-10 rating scale, 39% (CI 32-47%) estimated the likelihood of another pandemic (with another pathogen) to be *strictly higher* than that of a second wave of COVID-19. Vice versa, only 23% (CI 17-30%) estimated the likelihood of a second wave of COVID-19 to be *strictly higher*.

Table 6 shows how strictly the participants claimed to be following certain preventive guidelines *at the time of the survey* (A1-A7 in Table 6). Like Table 5, the upper part of Table 6 is split in its middle, such that the left side of the table cumulates frequencies from high to low likelihoods, starting on the left with the highest (“always”), and the right side of the table cumulates frequencies from low to high likelihoods, starting from the right with the lowest (“never”). Strict adherence (answer option “always”) was most frequent regarding coughing and sneezing only into a tissue or the inside of one’s own elbow (89%; 97% at least “almost always;” A6), not shaking hands (82%; 96% at least “almost always;” A5), and not leaving home in case of a cough or fever and contacting the hotline or a physician via phone (81%; 89% at least “almost always;” A7). 56% (75% at least “almost always”) claimed to always refrain from public transportation during rush hour (A1), while 8% did not refrain from public transportation during rush hour at all. 36% (67% at least “almost always”) disinfected or washed their hands with soap after each physical contact (except with family, A4). Only 8% were able to always (50% at least “almost always”) keep a physical distance of at least two meters all the time (except their closest family, A3), which is not surprising, given that all of the participants regularly worked with patients. For each of the five covered preventive guidelines, the proportion of participants who followed them at least “predominantly” lay above 80% (CIs above 74%). Participants in leading positions were *more* likely to refrain from public transportation during rush hour (at least “almost always,” AME = 18.5 percentage points, *p* < 0.01, binary logistic regression), participants living by themselves were *less* likely to keep a physical distance of two meters from people except their closest family (at least “almost always,” AME = –33.7 percentage points, *p* < 0.001), and participants who were part of a risk group regarding COVID-19 because of their health condition were *more* likely to disinfect or wash their hands with soap after each physical contact (excepting their family) (at least “almost always,” AME = 19.4 percentage points, *p* < 0.05).

The lower part of Table 6 shows for how long the participants expected to continue to follow the guidelines with the same intensity *in the future*, that is, following the survey. The following proportions of participants expected to continue indefinitely or until a vaccine would be available: 92% with coughing and sneezing only into tissue or inside their elbow (A16), 55% with disinfecting or washing their hands with soap after each physical contact (except with family, A14), 47% with not leaving home in case of a cough or fever and contacting the hotline or a physician via phone (A17), 45% with not shaking hands (A15), 35% with not using public transportation during rush hour (A11), and 31% with keeping a physical distance of at least two meters from everyone except their closest family (A13). While not leaving home in case of a cough or fever and not shaking hands were both followed with high adherence at the time of the survey, roughly half of the participants expected to keep it up for a year or less only, and to not necessarily wait until a vaccine would be available. These two guidelines concern socially and culturally relevant behaviors. Staying at home may be perceived as an act of social isolation, depending on the situation, and shaking hands is a common gesture of greeting in Switzerland. Refusing an offered handshake without providing a reason, such as a health hazard, can be considered as a sign of disrespect. The analysis of those participants who claimed to adhere to the guidelines at least “predominantly” at the time of the survey showed that participants of age 45 to 54 were *more* likely to continue keeping a physical distance of two meters until a vaccine would be available (AME = 24.6 percentage points, *p* < 0.01), and that participants of age 55 and above were *even more* likely to continue keeping a physical distance of two meters (AME = 42.0 percentage points, *p* < 0.001), with both age groups being compared to participants of age below 45. Furthermore, participants who had passed the majority of their education outside of Switzerland were *more* likely to continue disinfecting or wash their hands (AME = 27.5 percentage points, *p* < 0.01). Finally, participants of age 55 and above were *more* likely to continue not shaking hands (AME = 25.6 percentage points, *p* < 0.01), and participants who answered the survey on 20th June or later (see section “Data Analysis” for explanation) were *more* likely to continue not shaking hands (AME = 27.5 percentage points, *p* < 0.01).

Table 8 lists the pair-wise rank correlation of the reported adherence to the guidelines. Within each cell of the table, the upper coefficient refers to adherence *at the time of the survey* (A1-A7), and the lower coefficient refers to continued adherence *in the future following the survey* (A11-A17). Correlation across the different guidelines was rather low *at the time of the survey*. Even though mostly significantly different from zero, the effects were of *small* or *moderate* size according to the classification by Cohen (1992), except for the two pairs of A3/A4 and A3/A5. This means that an individual typically did not follow all guidelines to a uniform extent, but instead differentiated between the guidelines, and followed some of them more strictly and others less strictly. By contrast, correlation was high among continuation in *in the future*. Here, the effects were mainly *strong*, with coefficients up to 0.707, and only a few of them were moderate (those involving A16, which is the dimension with the highest expected *future* adherence by a large margin). Hence, an individual typically differentiated her/his behavior across the guidelines initially, and then intended to continue the pattern for a certain duration, without strongly readjusting it over time by relaxing on a part of the guidelines earlier than on others. Please note that the correlations regarding continuation *in the future* (A11-A17) were calculated for the subsample of the 95 participants who did not answer with “don’t know.” If the correlations regarding adherence *at the time of the survey* were computed for the same subsample (*n* = 95), the effects were even smaller than the ones shown in Table 8 (all but two of them).

Of the mentioned preventive guidelines (as listed in Table 6), two participants (2%, CI 1-5%) claimed that “most of them are exaggerated for persons working with patients or elderly people,” and 14% (CI 9-19%) claimed that “most of them are exaggerated for people *not* working with patients or elderly people.”

Figure 3 depicts the participants’ plans of traveling abroad before the end of the year 2020. Had the pandemic not emerged, 83% (CI 76-87%) would have traveled abroad. Given the pandemic, only 31% (CI 25-38%) still had plans of traveling abroad at the time of the survey. Unsurprisingly, participants who had passed most of their education in Germany (rather than in Switzerland) were *more* likely to still have plans of traveling abroad given the pandemic (AME = 44.2 percentage points, *p* < 0.001, binary logistic regression). One participant commented that she/he had elderly relatives abroad and therefore had to follow a “familial obligation.”

### Impact on Work Situation

Table 7 shows the participants’ assessment of the initial preparation for a viral pandemic before the outbreak (items P1-P5), how COVID-19 had affected their work situation (W1-11), and which lessons should be learned from its first wave (L1-L10). The participants largely indicated that before the COVID-19 pandemic had broken out, the preparation by the government and the healthcare sector for a viral pandemic had been insufficient. 91% deemed preparation insufficient regarding the availability of disinfectant and protective masks (P1), 86% regarding personnel (P2), 77% regarding structures (P3), and 70% regarding processes and contingency plans (P4). More than half of the participants (58%, CI 51-65%) claimed that in none of these four areas preparation had been sufficient (P5).

Following the outbreak, 44% of the participants felt more stressed than usual because of the pandemic (W1 in Table 7). 38% worked unusual tasks as a result of the COVID-19 pandemic (W4), and 32% worked more hours than usual (W2). 28% indicated that not all materials and structures necessary to effectively protect the healthcare staff from an infection with COVID-19 were available (W7), and 19% thought that not all the decisions necessary to do so were being taken (W8), respectively. 92% (CI 88-95%) of the participants reported multiple effects of the pandemic on their work situation (W1-W10). Only one participant concluded that the first wave of the pandemic had no effect on her/his work situation at all (W11). If a participant selected the item labeled “other” (W10), they were asked to specify these other effects. Among these text answers (*n* = 18), the most frequently mentioned issue was the handling of visitors of patients (four mentions), which grew more challenging due to more restrictive preventive measures and visitor hours, as well as due to visitors not abiding to them and even verbally abusing the staff. Three participants again emphasized a severe lack of protective equipment, one of them described “chaotic” circumstances, in which masks had been forbidden to be used by nurses until the first confirmed case had occurred within the institution, and with no measures of isolation afterwards. Three times it was claimed that wearing the protective material, particularly masks, made work more difficult or more exhausting. Three reports were given of increased psychological strain among the staff and the patients. Another three statements were made that organizational challenges were high, because changes needed to be implemented within very short time and without a test run. Single mentions were the introduction of tracking, a lack of personnel, economical aspects dominating the healthcare system, and employers threatening employees with consequences in case they should introduce COVID-19 into the institution. One participant reported to actually have less work because fewer patients were present in her/his institution due to the pandemic.

### Reaction by the Government

The vast majority of 72% (CI 65-78%) found the preventive measures implemented by the federal government between 17th March and 26th April 2020 (i.e., the “lockdown” during the first wave) to be “adequate.” Another 17% (CI 13-23%) found them to be “not strict enough / too late / too short in duration,” and 10% (CI 7-15%) found them to be “exaggerated.” 56% (CI 48-63%) concluded that the relaxation schedule from 27th April onward was “adequate,” while 32% (CI 26-39%) would have preferred the preventive measures to be relaxed “later / less strongly,” and 11% (CI 8-17%) claimed that the measures should have been relaxed “earlier / more strongly.” The above-mentioned date of 19th June (see section “Data Analysis”) was predictive of the evaluation the participants made. Participants who completed the survey after that date were significantly *more* likely to deem the relaxation plan as too liberal (i.e., relaxation should be done “later / less strongly”), compared to participants who completed the survey up to 19th June (AME = 0.281, *p* < 0.001, binary logistic regression). In addition, participants who had children were *less* likely to evaluate the relaxation plans as too liberal (AME = –0.185, *p* < 0.01), and participants who had passed the majority of their education in Germany were *more* likely to evaluate them as too liberal (AME = 0.285, *p* < 0.01).

### Key Lessons

More than half of the surveyed healthcare workers (58%, CI 51-65%) claimed the need for more/better medical equipment (including drugs) than it was available during the first wave of the COVID-19 pandemic (L4 in Table 7). 40% required better protection of their own physical health (L7), and even 44% called for better protection of their mental health (L8). 37% asked for more (assigned) personnel (L2). 37% thought that hourly wages should be higher due to the exceptional circumstances (L6). 36% required more detailed/accurate information about the COVID-19 symptoms (L3), and 32% called for an earlier warning next time (L1). Only 14% indicated that the work schedule should be left unchanged due to the pandemic (“business as usual,” L5). 7% claimed that no lessons needed to be learned, as preparation for and handling of the pandemic had been appropriate in their view (L10).

### Presumed Cause of the Pandemic

Half of the participants (54%, CI 46-61%) identified negligent behavior of humans towards animals/nature as the cause of the COVID-19 pandemic, as depicted in Figure 4. Six participants (3%, CI 1-7%) concluded that it was instead a willful transfer to humans as a biological attack. Among “other causes” (4%, CI 2-8%), mutation of SARS, improper hygiene in the food sector, politics, economics, overpopulation of the planet and overconsumption of natural ressources, ignorance, and denial were specified.

## Discussion

### Key Findings

This survey explored the knowledge of Swiss healthcare workers on COVID-19, how the first pandemic wave impacted their work situation, and how they reacted both emotionally and regarding their adherence to preventive guidelines.

Assessed after the first wave of COVID-19 had been overcome, clinical knowledge of COVID-19 was high among healthcare workers on several main topics, but not on all of them. In particular, a large proportion (more than a third) overestimated the effectiveness of standard hygiene (namely frequent washing of hands and sneezing into tissues) as a regime that would virtually exclude any transmission of COVID-19. This proportion was even higher among those who had worked more hours than usually during the pandemic. This misjudgment was prevalent, despite most of the respective healthcare workers knowing that COVID-19 was not only transmitted via physical contact. Also, and this may be critical, the vast majority of them nevertheless believed not to be needing any further information on the topics of preventive measures and transmission. Another topic where knowledge was limited, however to a lesser degree, was the comparison of COVID-19 with the common flu regarding infectiousness, incubation time, and life-threatening disease progression. Again, a pattern showed according to which the majority of those participants who did not provide the correct answer believed not to be needing any further information (except for incubation time, where the proportion was slightly smaller than half). This clearly shows that even after the first wave of the pandemic, healthcare workers had still not received comprehensive or uniform education on certain essential topics. It also reflects the circumstance that COVID-19 had not only been present in media of specific focus and readership, such as scientific media from which to be absorbed by the healthcare institutions, but that it had also been dominating the popular media since shortly after the outbreak. In this ever-present flow of information from most heterogeneous outlets, the distinction of scientific facts, or also a lack of scientific facts when it was the case, from speculation and opinion became significantly more challenging (see e.g., notion of *infodemics*, Lexico dictionary, 2020). This raises the question of by whom, and through which processes, the provision of comprehensive and uniform clinical information to healthcare workers can and should be ensured when managing a pandemic of global relevance. According to the healthcare workers, they most often expected the government to provide them with the necessary information, followed by scientists/universities, and their employer. Any other possible sources (e.g., journalists) should play a smaller role according to them. They preferred to receive the information by public television (and to a slightly lesser extent by radio and newspaper articles). In case the employer should provide them with according information, they had a clear preference for it to be in writing rather than orally.

The healthcare workers reported considerable emotional distress caused by the pandemic, with more than half of them feeling worried about their family or friends possibly getting infected, and about numerous deaths among elderly and sick people, respectively. About one in five reported to be feeling very worried because of these possibilities, while less than ten percent were not worried at all. By contrast, they were significantly less worried about themselves possibly contracting the disease. They were also asked to estimate the threat COVID-19 posed to different groups, irrespective of preventive measures, meaning for the hypothetical case in which no other precautionary measures would have been taken than the usual ones against the common flu. Again, they were significantly more concerned about the global and Swiss population than about themselves. Interestingly, they were also significantly more concerned about healthcare workers working with COVID-19 patients than about themselves. The latter was true even among healthcare workers who themselves attended to COVID-19 patients. While this finding may appear as a paradox, it is in line with the repeating pattern of them being more worried/concerned about others than about themselves, even if they are in the same situation. Even though this manifests as an altruistic trait, which may be lauded as “heroic” by society or patients (Cox, 2020), it ought not to be forgotten that this attitude serves the short-term interest of the patients, but could be detrimental to the physical and mental health of the healthcare worker.

The vast majority of the healthcare workers (three in four) estimated another wave of COVID-19 in Switzerland, after the first one that took place in March/April 2020, to be “rather likely.” A different pathogen causing another pandemic of equivalent or greater magnitude than COVID-19 within the next 20 years was considered to be even more likely. This provides the relatively clear picture that healthcare workers expected global pandemics to repeatedly be a part of human society in the future, and not a once-in-a-lifetime event.

The self-reported adherence to preventive guidelines was such that at least four in five healthcare workers followed them at least “predominantly.” The guidelines of refraining from shaking hands, no uncovered coughing or sneezing, and staying at home in case of a cough or fever, were followed strictly (meaning “always”) by at least four in five healthcare workers. All of the tested guidelines were official recommendations by the Swiss government during the “lockdown” phase of the first wave (however not legally binding, and relaxed after the “lockdown”). Interestingly, the pair-wise correlation across these guidelines was insignificant to moderate (with two exceptions), meaning that most healthcare workers displayed a pattern in which they did not follow all guidelines with the same commitment. Only between roughly a third and half of the healthcare workers expected to continue their pattern of adherence until a vaccine would be available in case that this would take longer than a year. This excluded the guideline of only covered coughing and sneezing, where the overwhelming majority expected to keep their adherence until a vaccine would be available (without a time limit). With increasing age, healthcare workers were more likely to expect to keep their adherence to both social distancing (two meters) and hand hygiene for a longer period of time. After eight in ten healthcare workers had plans of traveling abroad before the pandemic emerged, three in ten still kept such plans after the first wave.

The overwhelming majority of the healthcare workers stated, that the preparation by the government and the healthcare sector for a viral pandemic had been insufficient at the time COVID-19 emerged, especially regarding the availability of disinfectant and protective masks (nine in ten), but also clearly so regarding personnel (six in seven), structures (four in five), processes, and contingency plans (seven in ten). The majority even claimed that preparation had been insufficient in all of these areas. It is therefore not surprising that the reported effects of the pandemic on the work situation of the healthcare workers were rather diverse. Roughly one in three had worked more hours than usual. This finding was confirmatory of Spiller et al. (2020), who further found that hours worked were sluggish in converging back to previous levels. Even before the pandemic, excessive labor of healthcare workers had been an often-discussed topic in the literature, particularly regarding its effect on psycho-social function, productivity, and working errors in an industry, where the margin for error often is small (see e.g., Caruso, 2006; Griffiths et al., 2014). Another one in three healthcare workers had worked usual tasks. One in four reported that not all materials and structures necessary to effectively protect the healthcare staff from an infection with COVID-19 were available during the first wave. One in six (each) were more pressed for time, had an employer showing less consideration for their needs than usual, or observed a relevant share of nurses not strictly abiding to the hospital-/institution-specific regulations regarding protective masks, washing of hands, and physical distancing, respectively. Further, less frequently named effects were working for another department/division, challenging situations with visitors of patients due to increased precautionary measures (and some visitors not abiding and even being verbally abusive), physical exhaustion due to wearing a mask while working, increased pressure by the employer, increased psychological strain, and implementing new processes within short time and without testing. The most frequently reported effect, however, was an increase in emotional stress level as a result of the COVID-19 pandemic (almost half of the healthcare workers).

The vast majority of the healthcare workers found the reaction by the Swiss government, specifically the “lockdown” during the first wave, to be adequate, while one in six found it to be not restrictive enough (or too late/short), and one in ten found it to be exaggerated. The relaxation plan following the “lockdown” received significantly less approval, with one in three healthcare workers claiming that the preventive measures should have been relaxed later (or less strongly), and one in ten claiming the opposite. The policy change announced by the national government on 19th June, according to which many restrictive measures would be relaxed or abolished, the national coronavirus taskforce (KSBC) would be suspended, and the management of further pandemic waves in the future would be mainly the duty of the cantons, was deemed as too liberal by a significant proportion of healthcare workers. A similar result showed in the analysis of their adherence to preventive guidelines, in which the healthcare workers who participated in the survey after this change of policy were significantly more likely to expect to continue not shaking hands at least until a vaccine was available, compared to healthcare workers who had participated before this change of policy.

### Lessons to Be Learned

Key lessons were drawn which should be learned according to healthcare workers themselves. They should be seen as recommendations for the management of further pandemic waves which have recently developed in Switzerland and many other countries.

According to the surveyed healthcare workers, the lesson most often claimed as needed to be learned was the requirement of more/better medical equipment (including drugs) than during the first wave. This again reflects the lack of protective materials at the beginning of (and also during) the first wave in Switzerland, as well as the globally ongoing efforts in research for vaccination and therapeutics. This can be seen as the first aim of improvement according to healthcare workers. While their personal physical and mental wellbeing, as well as their ability to fulfill their tasks effectively and efficiently, are affected by other factors as well, progress towards this first aim can be expected to yield most significant improvement. The healthcare workers’ second priority was better protection for their own mental and physical health (with mental health being named more frequently, however with a statistically insignificant difference compared to physical health). A proportion of more than four in ten stated this need. This is in accordance with the above-mentioned group of medical organizations, which together recently issued an open call to the Swiss government for support in order to prevent further deterioration of the state of Swiss healthcare workers (see section “Introduction”). In addition to practical challenges, a viral pandemic can cause a moral dilemma of being responsible for patients, but thereby also risking getting infected and infecting others, which may impose additional mental and emotional strain and even affect decision-making. Irrespective of the COVID-19 pandemic however, the literature has suggested that healthcare workers find themselves in a difficult industry, as far as emotional, communicational, and decision-making challenges are concerned (see e.g., Wulf, 2012; Joseph and Joseph, 2016), which can be psychologically depleting. In this sense, the COVID-19 pandemic can be seen as an event which has not only caused new challenges for healthcare workers, but which has also emphasized shortcomings that were prevalent beforehand. Solutions therefore should address both the pandemic-specific as well as the underlying long-term challenges of the industry. The third lesson was the need for more personnel to be available (and assigned) to handling the pandemic, as well as increased hourly wages during the exceptional circumstances. It needs to be kept in mind that during a pandemic, healthcare workers getting infected themselves is a twofold risk, as it not only threatens the health of the individual, but also isolates her/him from the workforce at least for a period of quarantine. Fourthly, more detailed information about the symptoms of the disease was required, as well as a system of earlier warning in order to provide room for preparation. Each of these lessons were named by more than three in ten healthcare workers (some significantly more). Nevertheless, there was a small minority of healthcare workers (one in fifteen), who claimed that no lessons needed to be learned from the first wave of the pandemic, as preparation for and handling of it had been appropriate in their view. Given all of these results, the fifth lesson to be learned is that healthcare workers and their individual situations are considerably heterogeneous. They have faced a variety of different consequences and challenges during the pandemic, and some have been affected more strongly than others. Therefore, solutions must be specific to varying circumstances and remain adjustable over time.

### Limitations

The population of healthcare workers who directly attend to patients during the present COVID-19 pandemic is at the center of the topic. To date, no randomized sample with mandatory participation (or complete survey) has been drawn from this population in Switzerland. Therefore, clustered sampling was conducted for this survey, contacting the attendees of extra-occupational professional development courses at Careum Weiterbildung in Aarau. The vast majority of healthcare workers in Switzerland repeatedly attend such courses, and most of the institutions offering these courses follow a similar scheme. Careum Weiterbildung encompasses a wide range of attendees from different institutions, areas of healthcare, and geographical regions across Switzerland. The sample of this survey therefore was drawn from a very broad population of Swiss healthcare workers. It needs to be noted however, that participation was not mandatory within the cluster of Careum Weiterbildung. Therefore, randomness cannot be ascertained, nor excluded. Also, despite the teaching institutions being of a similar scheme, and despite the regions from which they attract students overlapping, homogeneity of the clusters is unproven. The sample size is limited. A larger sample, although not necessarily related to unbiasedness, could decrease the error probabilities on inferential statistical tests. Causal effects of the pandemic were assessed by directly asking the participants to do so themselves, whenever considered to be expedient, e.g., by asking “how has the COVID-19 pandemic affected your work situation?” Within the cross-sectional design of the study, concepts such as emotional distress and risk perception could not be tracked over time before/during the pandemic, as a panel or follow-up study could have. Moreover, all data was self-reported by the participants. Emotional distress was measured by four items. These were derived by three questions on how worried they were, as shown in Figure 2, referring to three different groups (/oneself) which the pandemic may threatened by the pandemic, with answer options on a four-point Likert scale. Also, the participants indicated whether they felt more stressed during work because of the COVID-19 pandemic, by answering a yes/no question (item W1 in Table 7). A seven-item validated scale of the fear of COVID-19 has been published by Ahorsu et al. (2020), which aims at differentiating emotions more strongly (feeling “afraid,” “uncomfortable,” “nervous,” having clammy hands, a racing heart, losing sleep) and could yield more detailed insight. Since this study was conducted for Swiss healthcare workers, understanding their specific situation at the time was crucial. Consequently, the findings may only be applicable to nations/healthcare systems, in which the first wave of the pandemic followed a comparable pattern.

## Data Availability Statement

The datasets presented in this article are not readily available because of requirements of anonymity. However, the raw data supporting the conclusions of this article will be made available by the authors to any qualified researcher, excluding the demographic data and the free text answers, such that any inference that would breach the anonymity of an individual remains ruled out. Requests to access the datasets should be directed to MR, marco.riguzzi@careum-hochschule.ch.

## Ethics Statement

Ethical review and approval was not required for the study on human participants in accordance with the local legislation and institutional requirements. Written informed consent for participation was not required for this study in accordance with the national legislation and the institutional requirements.

## Author Contributions

MR contributed to the quantitative methodology, data curation, and formal analysis. SG performed the literature research. Both authors contributed to the conceptualization, composing and online implementation of the questionnaire, writing and editing, project administration, and article. Both authors approved the submitted version.

## Conflict of Interest

The authors declare that the research was conducted in the absence of any commercial or financial relationships that could be construed as a potential conflict of interest.

## Footnotes

1 Average effective reproductive number over the last 3 days, as estimated by the Swiss Federal Institute of Technology (ETH) Zürich, which states: “Re values above 1 are not a consequence of an increased testing effort or of false positive test results. […] the percentage of positive tests among all tests (i.e., the test positivity rate) has increased from 0.4% in June to around 15% at the moment. When correcting for the increase in testing effort in the statistical analyses, we still estimate Re significantly above 1 for most of the summer. Additionally, the specificity of PCR tests is very high, leading to essentially no false positive results which could bias our estimates.” (ETH, October 27^*th*^ 2020). Description of the estimation method: https://ibz-shiny.ethz.ch/covid-19-re/.

2 Although participants could choose to name an e-mail address to which a message would be sent in the future providing information on where the results of the study would be published.

3 Some individuals worked in more than one area.

4 The initial set of predictors for which factor elimination was performed comprised the following items, for which one-sided causality could be assumed : W2 through W5 (see Table 7), health sector, specialized field, hierarchical level, gender, children, living by oneself, country, works with COVID-19 patients (see Table 1), education (none / healthcare assistant / nurse with regular diploma / nurse with diploma of Swiss “höhere Fachschule, HF” / Bachelor’s degree in nursing / Master’s degree in nursing / other), health condition making the individual part of a COVID-19 risk group, answered the questionnaire before 20th June 2020 (see the final paragraph of the “Data Analysis” section for explanation). In some cases, minimization of BIC led to a reduction of the model to a single predictor, as reported in the “Results” section.
